# Remodeling of Intrahepatic Ducts in a Model of Caroli Syndrome: Is Scar Carcinoma a Consequence of Laplace’s Law?

**DOI:** 10.3390/medsci7040055

**Published:** 2019-04-01

**Authors:** Bharvi M. Chavre, Kai Jiang, Luce G. St. Surin, Terrence Bissoondial, Ping Zhou, Jingsong Li, Satishkumar V. Gadhiya, Itzhak D. Goldberg, Prakash Narayan

**Affiliations:** Department of Preclinical Research, Angion Biomedica Corp., Uniondale, NY 11553, USA; bchavre@gmail.com (B.M.C.); kjiang@angion.com (K.J.); lstsuri1@jhu.edu (L.G.S.S.); bissoondialt@gmail.com (T.B.); pzhou@angion.com (P.Z.); jli@angion.com (J.L.); sgadhiya@angion.com (S.V.G.); igoldberg@angion.com (I.D.G.)

**Keywords:** Caroli syndrome, congenital hepatic fibrosis, intrahepatic bile duct, scar, hepatocellular carcinoma, cholangiocarcinoma

## Abstract

Caroli syndrome, characterized by saccular dilatation of intrahepatic ducts and congenital hepatic fibrosis, is without therapy in part due to its ultra-rare prevalence and the apparent lack of availability of a suitable experimental model. While the PCK rat has long been used as a model of fibropolycystic kidney disease, hepatobiliary biophysics in this animal model is incompletely characterized. Compared to age-matched, wild-type controls, the PCK rat demonstrated severe hepatomegaly and large saccular dilated intrahepatic ducts. Nevertheless, hepatic density was greater in the PCK rat, likely due to severe duct wall sclerosis accompanied by scarring across the hepatic parenchyma. Extracellular matrix accumulation appeared proportional to duct cross-sectional area and liver volume and appeared compensatory in nature. The PCK rat livers exhibited both cholangiocarcinoma and hepatocellular carcinoma coincident with areas of increased extracellular matrix deposition. Together, these data suggest that the PCK rat model mimics at least in part the spectrum of hepatobiliary pathology observed in Caroli syndrome and highlights the attendant risk associated with this disease.

## 1. Introduction

Caroli syndrome (CS) is characterized by non-obstructive saccular dilatation of large intrahepatic bile ducts, accompanied by congenital hepatic fibrosis (CHF) [1,2]. Quite often, CS is associated with autosomal recessive polycystic kidney disease (ARPKD) [3]. Left untreated, pathology in CS is progressive and can lead to clinical complications including frequent episodes of bacterial cholangitis, formation of bile stones, portal hypertension, hepatobiliary degeneration, and cholangiocarcinoma [4,5,6,7]. Hepatic transplantation is indicated once severe cholangitis, malignant transformation, or even suspicion of malignancy is present. In CS with ARPKD, combined or sequential hepatorenal transplantation is indicated [8,9,10,11]. Limited pharmacological options in CS are driven, at least in part, by both its ultra-rare nature [12], and the lack of availability of a model that recapitulates the spectrum of clinical hepatobiliary findings. 

The PCK (PCK/*CrljCrl-Pkhd1^pck^/Crl*) rat is a model of ARPKD [13,14]. Although the majority of studies using this model pertain to the fibrocystic renal disease, there are a few reports of ductal plate malformation, enlarged bile ducts, and congenital hepatic fibrosis in these rats, suggesting that it might serve as a model of CS [15,16]. The present study performs a biophysical and histopathological characterization of hepatobiliary pathology in these animals. 

## 2. Methods

### 2.1. Animal Models 

The animal protocol was reviewed and approved (10 March 2016) by the Angion Biomedica Corp., NY, USA animal care and use committee (IACUC approval, 2016-004). Animals had access to food and water *ad libitum* throughout the course of the in-life protocol. Male PCK/*CrljCrl-Pkhd1^pck^/Crl* (*n* = 14) and Sprague–Dawley rats (*n* = 11, wild-type (WT, control)) were administered a standard laboratory diet and water ad libitum. At ~7 months of age, animals were sacrificed and blood and liver retrieved for analysis.

### 2.2. Liver Function Test and Biochemical and Histopathological Analysis

Liver and body mass were measured at sacrifice. Serum samples were sent to Northwell Health laboratory, NY, USA for measurement of aspartate aminotransferase (AST) and alanine aminotransferase (ALT), whose levels were expressed in International Unites (IU). Liver volumes were recorded by fluid displacement and liver density calculated as the liver mass to liver volume ratio. A portion of the liver was retrieved and snap frozen in liquid N_2_ for measurement of hydroxyproline (mg/liver), a marker of tissue scarring. Intrahepatic duct cross-sectional area, expressed as a percentage of parenchymal field area, was quantified in hematoxylin and eosin (H&E)-stained slides using digital planimetry (BIOQUANT, Nashville, TN, USA) or ImageJ (National Institutes of Health, Bethesda, MD, USA). Hepatic fibrosis, including collagen accumulation across the biliary wall, was visualized and quantified (% parenchymal field) using Picrosirius Red (PSR) and Masson’s Trichrome staining. Multiple fields were photographed across each liver sample and data from those fields averaged for that liver. ImageJ software (National Institute of Health) was used to determine duct wall thickness using a previously calibrated grid. Steatosis, cholangiocarcinoma, and hepatocellular carcinoma diagnoses were made via microscopic observation by two trained and independent observers as previously reported [17]. To confirm cholangiocarcinoma and/or hepatocellular carcinoma, alpha-fetoprotein (AFP) [18] was detected in formalin fixed paraffin-embedded liver sections using 15 µg/mL chicken anti-rat AFP antigen affinity-purified Polyclonal Antibody (Catalog # AF1369) overnight at 4 °C, counterstained with Vector Blue (Cat. No. SK-5300, Vector Laboratories, Burlingame CA, USA) and observed under a microscope. Additionally, cytokeratin 7 staining (anti-Cytokeratin 7 antibody (RCK105), Abcam, San Francisco, CA, USA), which localizes to neoplasms [19], and carbohydrate antigen (Ca)-9-19 [20] staining (anti-Ca-19-9 antibody, orb27274, Biocompare, South San Francisco, CA, USA) were used to confirm the presence of liver cancers. 

### 2.3. Data Analysis

Data are presented as mean ± standard error or mean. Student’s *t*-test was used to compare data between groups. Microsoft Excel (version 2016, Microsoft, Redmond, WA, USA) curve fitting software was used to generate all scatterplots. Wherever a linear relation was observed between the two variables in each of the scatterplots, the Pearson product moment (*r*) was calculated from the trend line. To determine whether the relationship between the two variables was significant, r and the sample size were entered into an online calculator [21]. A *p* < 0.05 was considered to be statistically significant in all cases. 

## 3. Results

**Figure 1:**
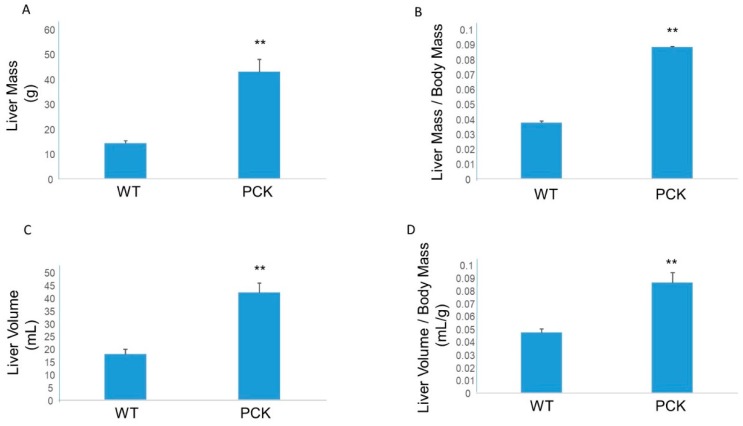
Hepatomegaly in the PCK rat. Compared to livers from the wild-type (WT) cohort, livers from PCK rats exhibited increased mass (**A**), increased liver to body mass ratio (**B**), increased liver volume (**C**), and increased liver volume to body mass (**D**). ** *p* < 0.01 vs. WT.

Compared to the wild-type cohort, livers from the PCK cohort exhibited significant hepatomegaly evidenced by increased liver mass, liver to body mass ratio as well as increased hepatic volume and hepatic volume to body mass ratio (Figure 1). In fact, these values were 2- to 3-fold greater in the PCK rat compared to WT.

**Figure 2:**
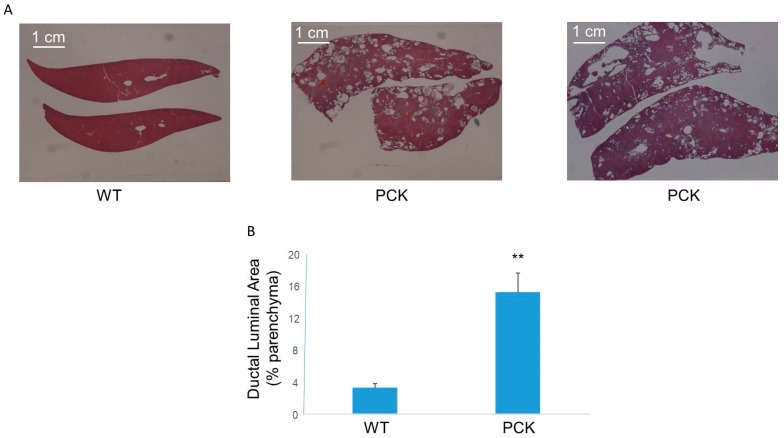
Hepatic phenotype in the PCK rat. (**A**) Representative photomicrographs (4×) of hematoxylin and eosin (H&E)-stained livers from WT and PCK rat showing multiple highly dilated intrahepatic bile ducts in the latter. (**B**) Measurement of ductal luminal area showed an ~5-fold increase in this parameter compared to the WT cohort (** *p* < 0.01 vs. WT).

Examination of H&E-stained sections of livers revealed large ultramicroscopic dilatations of intrahepatic bile ducts (Figure 2). Measurement of intrahepatic duct luminal area showed an ~5-fold increase in cystic index compared to the WT cohort (Figure 2). 

**Figure 3:**
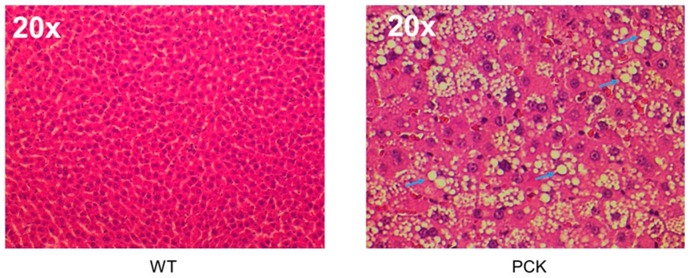
Steatosis in the PCK rat. Representative photomicrographs (20×) of H&E-stained livers from WT and PCK rats showing macrovesicular steatosis in the latter. Numerous lipid droplets (blue arrows) are evident throughout the hepatic parenchyma of the PCK rat.

Additionally, 11 out of the 14 PCK animals exhibited steatosis, evidenced by the presence of lipid droplets in H&E-stained liver sections (Figure 3).

**Figure 4:**
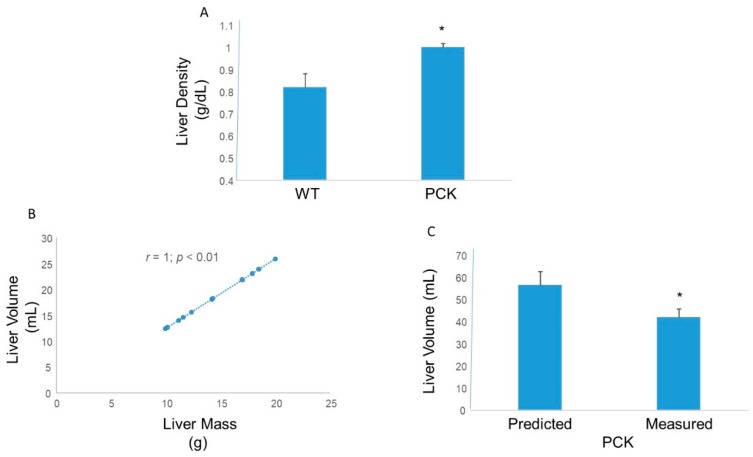
Hepatic density in PCK rats. (**A**) Calculated liver density in the PCK rat was ~20% higher than its WT counterpart (* *p* < 0.05 vs. WT). (**B**) Regression analysis between liver mass and liver volume in WT rats showed an excellent correlation. Liver volume increased linearly as a function of liver mass in at least the range of liver masses studied. (**C**) The regression line was used to predict liver volume in the PCK cohort. Predicted liver volume was significantly higher than the measured (liquid displacement method) liver volume in the PCK rat (* *p* < 0.05 vs. predicted).

Despite the presence of multiple large intrahepatic cysts, and lipid droplets, hepatic density, expressed as liver mass over liver volume was significantly greater in the PCK rat (Figure 4). This finding was confirmed using a training set comprising WT animals wherein liver volume was plotted against liver mass (Figure 4). This correlation yielded the following relation:

(1) y=1.347x−0.9678,

where *y* = liver volume (mL) and *x* = liver mass (g).

Based on this relation, in the PCK rat, the measured liver volume was significantly smaller than the predicted liver volume (Figure 4).

**Figure 5:**
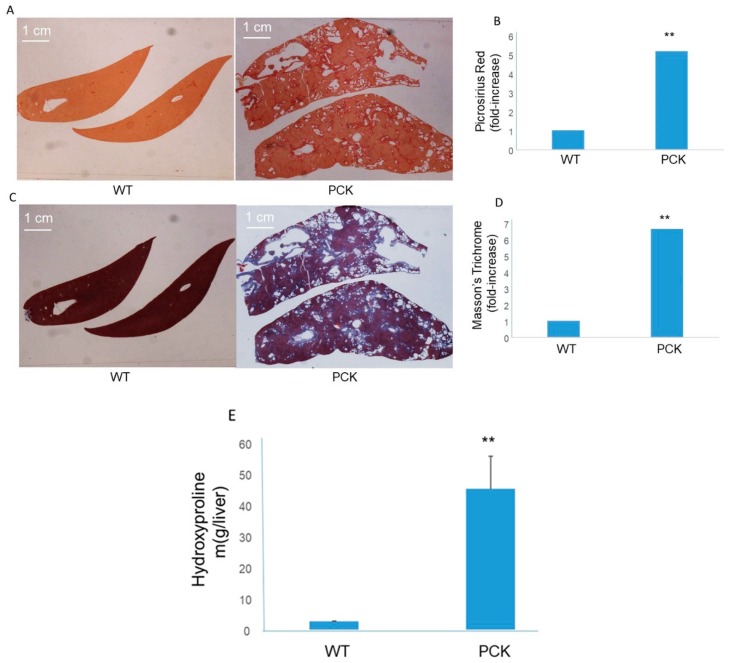
Liver scarring in the PCK rat. (**A**) Representative photomicrographs (4×) of Picrosirius Red (PSR)-stained livers from a WT and PCK rat. Staining was more robust in the PCK rat liver and appeared along the duct walls and as “bridges” across the hepatic parenchyma. (**B**) PSR staining was 5 to 6-fold more pronounced in the PCK rat liver vs. WT rat liver (** *p* < 0.01 vs. WT). (**C**) Representative photomicrographs (4×) of Masson’s Trichrome-stained livers from a WT and PCK rat. Staining was more robust in the PCK rat liver and appeared along the duct walls and as “bridges” across the hepatic parenchyma. (**D**) Staining was 6 to 7-fold more pronounced in the PCK rat liver vs. WT rat liver (** *p* < 0.01 vs. WT). (**E**) Compared to the WT cohort, PCK rat livers exhibited a significant increase in hydroxyproline measured in hepatic homogenates (** *p* < 0.01 vs. WT).

Liver sections stained with PSR from PCK rats were examined to determine the presence of scarring, if any, across the hepatic parenchyma. Compared to the WT cohort, PCK livers exhibited extensive scarring (Figure 5). Scarring was most pronounced along the intrahepatic ducts but bridging fibrosis was also evident across the entire hepatic parenchyma. The PSR signal was 5- to 6-fold elevated in the PCK livers compared to the WT livers (Figure 5). Masson’s Trichrome-stained livers exhibited a similar pattern of scarring, most pronounced across the hepatobiliary ductal walls with accompanying bridging fibrosis, and a 6 to 7-fold elevation in the blue (collagen) signal in the PCK livers vs. the WT cohort (Figure 5). Analysis of hepatic homogenates for hydroxyproline content (a marker of collagen) showed marked and significant elevation in the PCK livers (Figure 5). Liver function tests revealed no difference in ALT (36 ± 1.7 IU vs. 41 ± 3.7 IU, WT vs. PCK, respectively), but a mild elevation in AST (115.6 ± 7 IU vs. 177 ± 24.7 IU, WT vs. PCK, respectively; *p* < 0.05)

**Figure 6:**
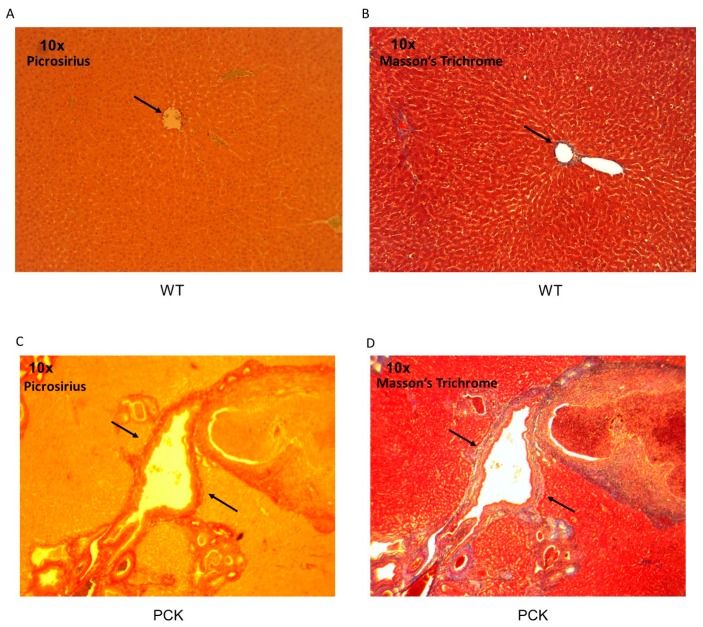
Intrahepatic ducts. (**A**) Photomicrograph of a typical intrahepatic duct (arrow) in a WT liver stained with PSR. (**B**) With Masson’s Trichrome staining, the intrahepatic duct wall (arrow) in a WT liver appears blue. (**C**) Photomicrograph of a typical highly enlarged intrahepatic duct in a PCK liver stained with PSR. Duct lumen is enclosed by a thickened wall (arrows) that is stained brightly with PSR, indicative of collagen accumulation. Staining extends into the hepatic parenchyma. (**D**) With Masson’s Trichrome staining, a similar pattern is observed with the collagen appearing blue (arrows).

Perhaps the most visually compelling feature of the PCK livers was intrahepatic duct remodeling evidenced by exaggerated intrahepatic ducts bound by thickened walls visible under PSR and Masson’s Trichrome staining (Figure 6).

**Figure 7:**
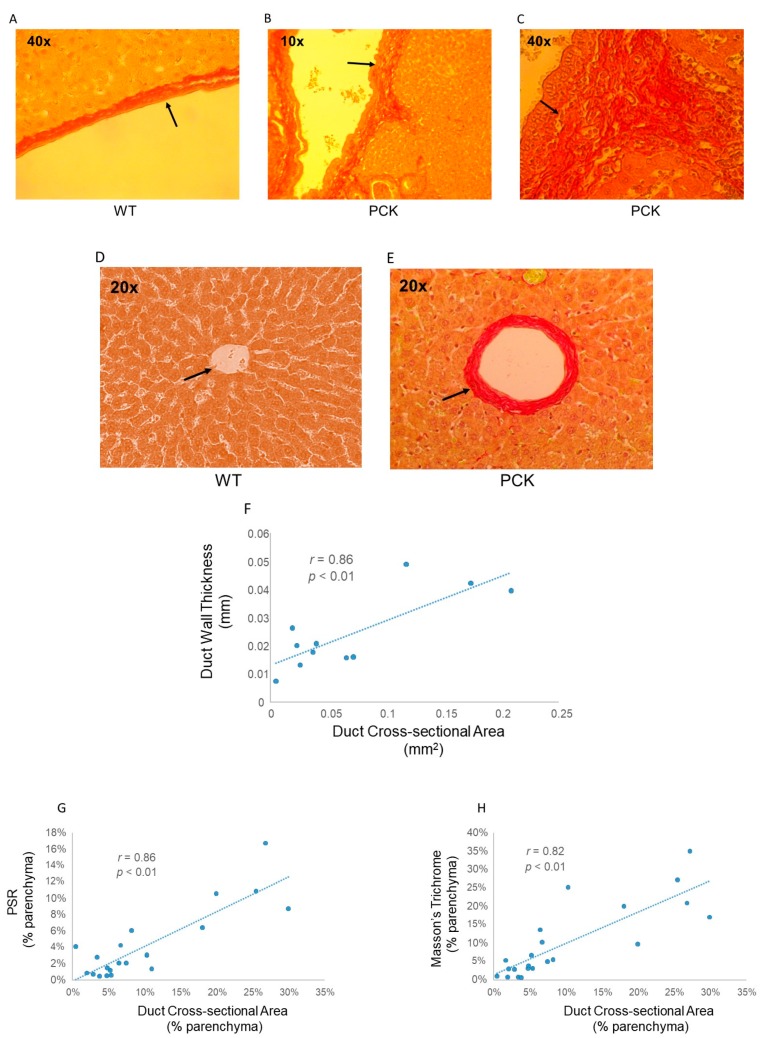
Intrahepatic duct remodeling. Representative photomicrographs of PSR-stained livers from WT rats (**A**, 40×) and PCK (**B** and **C**, 10× and 40×, respectively) rats. Matrix accumulation and duct wall thickening are far more pronounced in the PCK rat compared to WT. Duct wall thickness under 10× magnification (**B**) in the PCK rat liver exceeds that of the WT rat liver (**A**) observed under 40×. This difference is best appreciated when representative intrahepatic duct walls (arrows) from both a WT rat liver (A) and a PCK rat liver (**C**) are observed at 40×. Representative photomicrographs of PSR-stained livers from a WT rat (**D**), and a PCK rat (**E**) showing increased duct wall thickening accompanying increased duct cross-sectional area (arrows). Regression analysis (**F**) of duct cross-sectional area vs. duct wall thickening showed a direct correlation between the two. A direct and significant correlation between duct cross-sectional area vs. amount of scar (**G**, PSR staining, and **H** Masson’s Trichrome) is also evident.

Intrahepatic duct wall thickness appeared to increase with duct cross-sectional area. In fact, duct wall thickness in PCK livers observed under 10× magnification appeared as pronounced as duct walls from WT livers under 4-fold greater magnification (Figure 7). Under identical (40×) magnification, duct walls from PCK livers appeared multifold thicker compared to the WT cohort. Regression analysis of duct cross-sectional area vs. duct wall thickening exhibited excellent correlation (Figure 7). Expressed as a percentage of the parenchymal field under view, increased matrix deposition visualized using either PSR or Masson’s Trichrome staining also correlated with intrahepatic duct cross-sectional area (Figure 7). 

**Figure 8:**
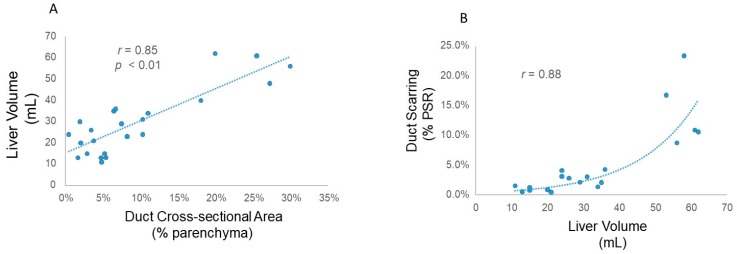
Liver remodeling in Caroli syndrome (CS). (**A**) A plot of intrahepatic duct cross-sectional area against liver volume in WT and PCK rats shows a strong and direct correlation between these two variables. (**B**) An exponential increase in duct wall scarring (PSR) with increasing hepatic volume is seen.

As a matter of fact, cross-sectional area of the intrahepatic ducts drove overall hepatic volume and as hepatic volume increased duct wall scarring (PSR staining) increased (Figure 8).

**Figure 9:**
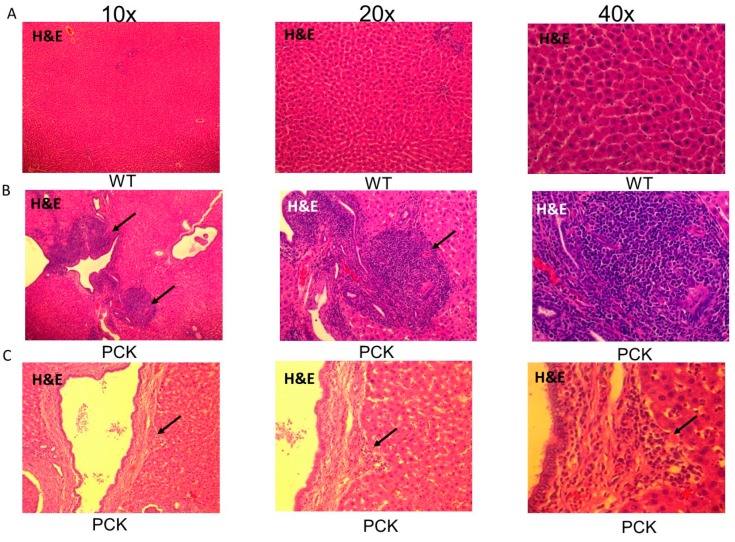
Scar carcinoma in the PCK rat liver. (**A**) Representative H&E-stained livers photomicrographs sections (10×, 20× and 40×) from a WT rat showing healthy parenchyma. (**B**). Representative H&E-stained livers photomicrographs sections (10×, 20×, and 40×) from a PCK rat showing trabecular growth pattern of atypical hepatocytes and clusters of multinucleated hepatocytes with a distinct margin (arrows) between the cancerous and non-cancerous parenchyma. (**C**) The hepatocellular carcinoma appears to arise from cholangiocarcinoma that is seen along the highly thickened intrahepatic duct wall in the PCK rat.

Another salient feature in the PCK rat was the presence of cholangiocarcinoma and hepatocellular carcinoma. While some PCK livers presented with tumors on gross observation itself, under the microscope most PCK livers exhibited multiple proliferating nuclei along the most thickened and scarred intrahepatic ducts (Figure 9). In many instances, the cholangiocarcinoma appeared to invade into the surrounding hepatic parenchyma. At many locations, the hepatic parenchyma presented with a trabecular growth pattern of atypical hepatocytes and clusters of multinucleated hepatocytes. A distinct margin (see arrows) was observed between the cancerous and non-cancerous parenchyma (Figure 9). 

**Figure 10:**
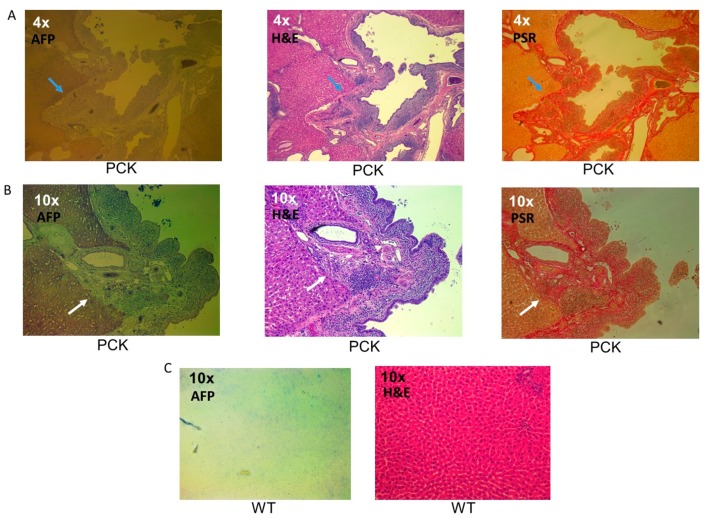
Cholangiocarcinoma in the PCK rat. (**A**) Representative liver photomicrograph (4×) from a PCK liver showing AFP (left) positive cells lining a duct. The cells have invaded the parenchyma. A similar view with H&E (middle) and PSR (right) staining demonstrating numerous proliferating nuclei and extracellular matrix deposition along the duct wall, respectively. (**B**) More detailed (10×) photomicrographs from the same PCK liver showing AFP staining (left), H&E (middle), and PSR (right), respectively. (**C**) Representative liver photomicrograph (10×) from a WT liver showing no AFP (left) positive cells. The H&E-stained (right) view is presented.

Presence of liver cancer, i.e., cholangiocarcinoma and/or hepatocellular carcinoma, was confirmed using the AFP antibody staining. Staining was localized and enriched in regions of ductal sclerosis and coincided with areas deemed cancer positive (H&E) and in areas with increased extracellular matrix deposition (Figure 10). Thirteen out of the 14 PCK rats presented with liver cancer, while all WT animals were cancer-free. 

**Figure 11:**
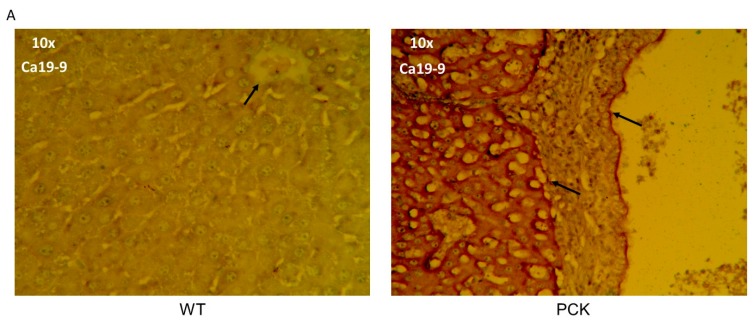

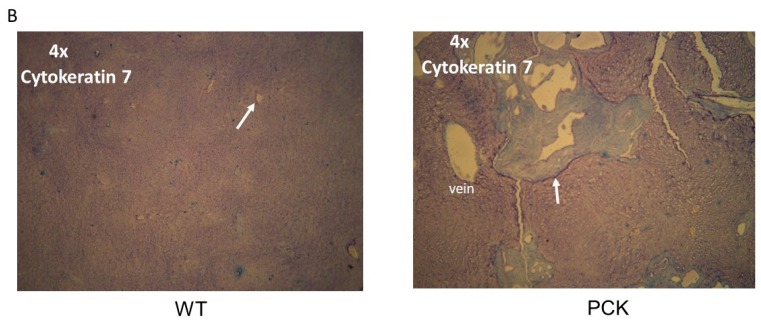
Cholangiocarcinoma and hepatocellular carcinoma in the PCK rat. (**A**) Representative liver photomicrograph (10×) from a Ca-19-9-stained WT liver (left) and PCK rat liver (right). The WT liver exhibits little or no Ca-19-9 staining (arrow shows an intrahepatic duct), whereas the PCK liver exhibits rich Ca 19-9 staining of the intrahepatic duct wall (arrow) and hepatic parenchyma (arrow). (**B**) Representative liver photomicrographs (10×) from cytokeratin 7-stained WT liver (left) and PCK rat liver (right). The arrows in each image point to the intrahepatic duct. The PCK liver exhibits rich cytokeratin staining of an intrahepatic duct but not a hepatic vein.

Further evidence of cholangiocarcinoma and/or hepatocellular carcinoma in this PCK model was evidenced by rich cytokeratin 7 staining restricted to intrahepatic ducts in the PCK rat (Figure 11) and corroborated by Ca19-9 staining of both intrahepatic ducts and hepatic parenchyma in the PCK but not WT cohort (Figure 11).

## 4. Discussion

Hepatobiliary pathology manifested by the PCK rat mimics in a large part CS in that it is characterized by hepatomegaly, large saccular dilated intrahepatic ducts, some steatosis, and ductal sclerosis due to extracellular matrix deposition accompanied by scarring across the hepatic parenchyma. Biophysical characterization of this rat model suggests that extracellular matrix accumulation appears proportional to duct cross-sectional area and liver volume. Livers exhibit both cholangiocarcinoma and hepatocellular carcinoma coincident with areas of increased extracellular matrix deposition. 

Treatment of CS remains a challenge for the pediatric gastroenterologist, with management of disease restricted to containing cholangial crises with use of a bile acid and antibiotics [4]. Liver transplantation is the therapeutic modality for decompensated organ failure, for prevention of malignancies arising from hepatocellular and/or cholangiocarcinoma, and in many cases for relief against progressive hepatomegaly [9,10,11]. Therapeutics to halt and/or reverse liver pathology have not been forthcoming. CS is an ultra-rare disease [12], with an estimated prevalence of 1/1,000,000 which translates to less than 500 patients within the USA. Clinical trials can therefore represent a major challenge within this population. Furthermore, even with the potential of premium pricing, large pharmaceutical companies have little interest in ultra-rare diseases. CS is often accompanied by more dominant and life-threatening pathology within the kidneys [6,8]. In ARPKD-CS, mortality in infancy is linked largely to pulmonary insufficiency—the very large renal volume precludes diaphragmatic movement and adequate expansion of the lungs [22]. Research efforts to combat disease have therefore centered around renal pathology [22]. Lastly, there is little if any description of experimental models that adequately mimic the spectrum of hepatobiliary findings in CS. 

Renal pathology in the PCK (PCK/*CrljCrl-Pkhd1^pck^/Crl*) rat, a model of ARPKD, has been extensively described by us and other investigators [13,14,23]. The autosomal recessive *Pkhd1* gene mutation in the PCK rat models human ARPKD. Relatively less is known about hepatic biophysics in this rat model. In fact, the relatively small number of studies [15,16], including two from this laboratory [14,23], evaluating liver pathology in this animal model have followed animals during the perinatal period or up until young adulthood, with only one report of study subjects being of 10 months of age [24]. Data from these published studies describe a model of Caroli disease accompanied by CHF-multiple segmental and saccular dilatations of intrahepatic bile ducts with dilatation encroaching the entire liver with age, and progressive overgrowth of portal connective tissue evident. Having previously reported on hepatic pathology in ~4-month-old PCK rats [14,23], we evaluated hepatic biophysics in ~7-month-old animals. At this age animals exhibited severe hepatomegaly, a finding consistent with clinical presentation of this disease. A striking feature of these livers were numerous and highly dilated intrahepatic ducts, clearly a progressive feature as previous studies from this laboratory did not report dilated intrahepatic ducts at ~4 months of age. Also not previously reported was steatosis within the majority of these ~7 months old PCK rats. This was a surprising observation given that these animals were fed standard laboratory chow and not a fatty diet. All the same, foci of micro- and macro-vesicular steatosis have been observed in human disease, suggesting that this animal model mimics that aspect of CS [25].

Another striking characteristic of this model was the scarring observed along the duct walls together with bridging fibrosis across the entire hepatic parenchyma. In fact, hepatic fibrosis is a hallmark feature of CS which distinguishes it from Caroli’s disease or saccular dilatation of the intrahepatic ducts without fibrosis. A major biophysical observation in this study is the increased scarring observed with increasing duct cross-sectional area and hepatic volume. In fact, several endpoints testify to this relationship. First, despite loss of solid parenchyma due to steatosis and large intrahepatic ducts, liver density was higher in the PCK rat compared to the WT rat. Second, ductal sclerosis tracked duct cross-sectional area, as did ductal scarring visualized by both PSR and Masson’s Trichrome staining. Furthermore, ductal sclerosis increased exponentially as liver volume increased. Together, these data suggest that the liver lays down extracellular matrix as a stabilization mechanism in the setting of expanding duct cross-sectional area (Figure 10). In fact, Laplace’s law of wall stress states that wall stress is inversely proportional to wall thickness and governs cardiac remodeling [26]. Increasing ductal sclerosis or wall thickening to stabilize the ballooning intrahepatic ducts from rupturing appears to be a direct consequence of Laplace’s law.

Another major phenomenological finding was cholangiocarcinoma and hepatocellular carcinoma in the PCK rat. To the best of our knowledge, this finding, albeit consistent with the increased risk for cancers in CS [5,6,7], has not been previously reported in the PCK rat. In many of the livers, the hepatocellular carcinoma appeared to arise from invading cholangiocarcinoma. Cancers were identified using four independent techniques: microscopy by a trained histopathologist, and use of antibodies to Cyokeratin 7, a marker of neoplasms, Ca 19-9, a carbohydrate antigen associated with gastrointestinal cancers, and AFP. A common finding regardless of methodology used was cholangiocarinoma and hepatocellular carcinoma in areas of increased ductal sclerosis and cholangiocarcinoma. This is best exemplified with AFP staining in which the AFP-positive region appeared to overlap with, or is at least localized to, regions of increased PSR staining/scarring/fibrosis. Although previously unreported in this mammalian model of CS, there is significant evidence for a continuum between fibrosis and cancer [27]. Cirrhotics are at increased risk for hepatocellular carcinoma [17]. In preclinical models of chemically-induced liver disease, including thioacetamide and CCl_4_-induced liver disease, animals develop liver fibrosis prior to hepatocellular carcinoma, an observation also shared in models of diet-induced liver disease [17]. Scar carcinomas have been previously reported in lung tissue [28,29]. While the exact mechanism underlying the transition from extracellular matrix deposition to cancer remains an area of intense investigation, it is also beyond the scope of the present report. Nonetheless, it does appear as if laying down extracellular matrix to support hepatobiliary structure leads to formation of carcinomas.

There were some limitations to this study. The data represent a snapshot in ~7 months old PCK rats. It could very well be that the transition to cholangiocarcinoma and hepatocellular carcinoma occurs earlier. Second, this is largely a phenomenological study. The exact mechanism underlying the formation of scar carcinomas remains to be delineated. Nevertheless, our findings are important in that if fibrosis is a requisite for cancer in CS, then drugs that interrupt or retard the fibrotic cascade might prevent or delay the formation of cancers. 

## 5. Conclusions

The PCK rat model presents with hallmark characteristics of CS. Liver pathology includes numerous highly dilated intrahepatic ducts, steatosis, and scar formation along the intrahepatic duct walls and across the hepatic parenchyma. In accordance with Laplace’s law of wall stress [26], scar formation might represent a mechanism to stabilize dilating ducts and a receding hepatic parenchyma. Scar formation is associated with cholangiocarcinoma and hepatocellular carcinoma. Biophysical and histopathologic characterization of the PCK rat model of CS might spur further research into development of therapeutics against this disease.
